# Utilization of acerola residue-derived biochars for methylene blue adsorption: effects of pyrolysis temperature

**DOI:** 10.1007/s11356-026-37397-5

**Published:** 2026-02-03

**Authors:** Jefferson David Oliveira da Silva, Sara Oliveira Santos, Antonio Martins de Oliveira Júnior, Ana Karla de Souza Abud

**Affiliations:** Centro de Ciências Exatas E Tecnologia, Universidade Federal de Sergipe, Aracaju, Brazil

**Keywords:** Acerola residue, Pyrolysis, Biochar, Adsorption, Aqueous effluents, Methylene blue

## Abstract

Adsorption is an effective method for removing various types of pollutants from aqueous effluents, and the production of adsorbent materials from biomass represents a sustainable alternative. In this study, biochars produced from the pyrolysis of acerola processing residues at temperatures ranging from 300 to 700 °C were evaluated as adsorbents for methylene blue dye. Batch adsorption tests revealed rapid adsorption kinetics, with equilibrium being reached within 30 min. The adsorption data were best described by the Langmuir isotherm and the pseudo-first-order kinetic model, indicating monolayer adsorption controlled by the availability of active sites. The biochar produced at 400 °C exhibited the highest adsorption performance, achieving a maximum adsorption capacity of 67.29 mg g^−1^ and a dye removal efficiency of approximately 94% at an initial concentration of 100 mg L^−1^. This superior performance was attributed to the optimal balance between surface area development and the preservation of oxygenated functional groups, which promote methylene blue adsorption through electrostatic interactions and hydrogen bonding. Overall, the results demonstrate the strong potential of acerola residue-derived biochars as low-cost and sustainable adsorbents for wastewater treatment, while also providing an environmentally sound destination for agro-industrial waste.

## Introduction

The pollution of water bodies due to the discharge of untreated effluents is a problem of global relevance (Tejada-Tovar et al. 2023). Among the various types of polluting compounds, organic dyes are commonly employed in several industrial processes, particularly in the textile industry, and their use generates a significant amount of contaminated wastewater (Ravindran et al. 2023). These substances are potentially toxic to aquatic life, and their improper disposal can also reduce sunlight penetration in water, hindering photosynthesis and negatively impacting the ecosystem (Li et al. 2016). It is estimated that annual dye production exceeds 700,000 tons, with approximately 12% of this total being discharged without treatment (Loke et al. 2022). Methylene blue is among the most widely used organic dyes, and the treatment of effluents containing this substance has recently been the focus of numerous studies (Al-Asadi et al. 2025; Das and Kundu 2025; Dasuki et al. 2025).

In this context, different technologies have been studied for the removal of organic dyes from wastewater before its final disposal. Due to its simplicity and effectiveness, the adsorption process is a promising alternative for removing these contaminants from aqueous effluents (Martínez et al. 2023). According to Shi et al. (2022), adsorption is a process in which molecules, atoms, or ions of a substance present in a fluid (gas or liquid) accumulate on the surface of a solid. Alternative materials, such as biochar, a product resulting from the pyrolysis of lignocellulosic biomass, have been extensively studied for dye removal applications, mainly due to their renewable nature and low cost (Ahmed et al. 2019).

The industrial processes of acerola juice (*Malpighia emarginata*), a fruit with increasing and significant commercial importance in Brazil, generate a large amount of waste that can cause serious ecological problems (Costa et al. 2024). The prospect of utilizing this biomass in biochar production is attractive from both economic and environmental perspectives, as it allows for the added value to the generated waste while reducing environmental damage (Silva et al. 2022).

Biochars possess surface characteristics that provide significant potential for use in adsorption processes. Nascimento et al. (2024) produced biochars through the pyrolysis of coffee agro-industrial waste and applied the resulting materials in the adsorption of the methylene blue dye. The authors reported that characteristics such as high specific surface area, the presence of various functional groups (carboxylates, carbonyls, hydroxyls, and amines), and a mesoporous structure provided the materials with an outstanding adsorption capacity for the pollutant, reaching approximately 41.98 mg g^−1^.

The pyrolysis temperature significantly affects these characteristics (Mahdi et al. 2017). In a previous study (Silva et al. 2022), it was observed that increasing the temperature led to biochars with greater porosity and increased surface area, but in a reduction in surface functional groups, such as hydroxyl and carboxyl groups, due to their thermal degradation. Chen et al. (2022) also observed a similar trend when analyzing the properties of biochars obtained from the pyrolysis of *Platanus orientalis* bark processed at different temperatures (200–600 °C).

Several studies have reported the use of biochars derived from different lignocellulosic residues for the adsorption of methylene blue dye, including pine sawdust and sugarcane bagasse (Luo et al. 2015), date seed (Mahdi et al. 2017), coconut shell (Nuryana et al. 2020), soybean dreg (Ying et al. 2021), rice straw (Bhardwaj et al. 2022), walnut green husk (Hou et al. 2023), and corn straw (Liu et al. 2024). These studies demonstrate that the adsorption performance of biochars is strongly governed by their physicochemical properties, such as surface area, pore size distribution, and the presence of oxygen-containing functional groups. However, these properties are not intrinsic to biochar itself but are largely dictated by the nature of the biomass precursor (Nascimento et al. 2024). The chemical composition, structural arrangement, and inorganic content of the original lignocellulosic material play a decisive role in determining the textural and surface characteristics of the resulting biochar and, consequently, its affinity toward dye molecules (Luo et al. 2015).

Despite the wide range of biomasses investigated, there is still a lack of studies exploring residues from acerola, a fruit of high agro-industrial relevance in Brazil. Additionally, most available studies focus on a limited number of pyrolysis temperatures, which restricts a systematic understanding of how thermal conditions affect biochar performance in dye adsorption.

In this context, the present work addresses this gap by producing biochars from acerola residue at a wide range of pyrolysis temperatures (300–700 °C) and systematically evaluating the effect of temperature on methylene blue adsorption. This approach allows not only the assessment of the adsorption potential of an unexplored biomass but also a detailed understanding of the structure–property–performance relationship, contributing to the development of sustainable adsorbents from agro-industrial waste.

## Materials and methods

### Preparation of the adsorbent

The processed acerola residues were provided by the company Duas Rodas, located in the Brazilian state of Sergipe. The biomass was dried at a temperature of 50 °C for 24 h in an oven to reduce moisture content (< 10%), and subsequently ground into particles between 0.5 and 1 mm using a Willye-type mill (Silva et al. 2020).

The biochars were produced from acerola agroindustrial residue following the procedure previously described by Silva et al. (2022), where it was applied thermochemical conversion. In summary, the biomass was pyrolyzed at temperatures of 300 (BC300), 400 (BC400), 500 (BC500), and 600 °C (BC600) in a rotary kiln reactor operating in continuous mode, while maintaining the following variables constant: a biomass feed rate of 1.5 kg h^−1^ with a total processing time of 20 min, a drum bed rotation at 7.5 rpm, a drum inclination angle of 10°, and an inert gas (N_2_) flow rate of 5 L min^−1^. For the present study, it was added an additional experiment conducted at 700 ºC (BC700) to support the assumptions regarding the adsorption process. Finally, the biochars were thoroughly washed using distilled water and then dried at 60 ºC in an oven.

### Biochar characterization

Biochar characterization was performed by proximate analysis, pH determination, elemental analysis, thermogravimetric analysis (TG/DTG), Fourier-transform infrared spectroscopy (FTIR), energy-dispersive X-ray fluorescence (EDX), and nitrogen adsorption–desorption isotherms for surface area and porosity evaluation. The detailed procedures and characterization data have been reported previously (Silva et al. 2022). In the present article, only the relevant results are discussed in relation to the adsorption performance.

### Adsorption assays

Batch adsorption experiments were conducted to evaluate the adsorption of methylene blue (MB). One hundred milligrams of adsorbents were placed in contact with 10 mL of MB solution at 10, 30, 50, 100, and 150 mg L^−1^, at times of 0, 5, 10, 15, 30, and 60 min. All experiments were carried out at room temperature (24 °C) and at the natural pH of the solution (pH ≈ 6).

Dye concentration was determined by measuring absorbance using an LGI Scientific model LGI-VS-721N UV–Vis spectrophotometer at 664 nm. The aliquots were centrifuged before analysis to remove the solid material. The adsorption capacity (*q*, mg g^−1^) and the removal percentage were calculated according to Eqs. 1 and 2, respectively.

1 $$q=\frac{\left({C}_{0}-{C}_{f}\right)V }{m}$$

2 $$\%\mathrm{removal}=\frac{\left({C}_{0}-{C}_{t}\right) }{{C}_{0}}100$$

where *C*_0_ is the initial concentration of the adsorbate solution (mg L^−1^ or g L^−1^), *C*_*f*_ is the final concentration of the adsorbate solution (mg L^−1^ or g L^−1^), *V* is the volume of the adsorbate solution (L) and *m* is the mass of the adsorbent used (g).

To evaluate the adsorption isotherms, the Langmuir model (Eq. 3), which assumes that adsorption occurs in a monolayer, i.e., each site can accommodate only one adsorptive entity (Langmuir 1918), and the Freundlich model (Eq. 4), an empirical equation based on multilayer adsorption, with non-uniform heat distribution (Freundlich 1906; El Haddad et al. 2014) were used.

3 $${q}_{e}=\frac{{q}_{\mathrm{max}}{k}_{\mathrm{L}}{C}_{e}}{1+{k}_{\mathrm{L}}{C}_{e}}$$

4 $${q}_{e}={k}_{F}{C}_{e}^{1/n}$$

where *q*_max_ is the maximum amount of monolayer coverage (mg g^−1^), *k*_L_ is the Langmuir isotherm constant (L mg^−1^), *C*_*e*_ is the adsorbate concentration at equilibrium (mg L^−1^), *k*_F_ is the Freundlich isotherm constant (mg L^−1^) (L g^−1^)^1/*n*^, 1/*n* is the heterogeneity factor and *q*_*e*_ is the amount adsorbed at equilibrium (mg g^−1^).

According to Hall et al. (1966), the essential characteristic of the Langmuir isotherm can be expressed by the dimensionless constant *R*_L_, called the separation factor and calculated by Eq. 5, allowing the shape of the adsorption isotherm to be predicted, indicating whether the adsorption is favorable or unfavorable.

5 $${R}_{\mathrm{L}}=\frac{1}{1+{K}_{\mathrm{L}}{C}_{0}}$$

To investigate the adsorption kinetics, the pseudo-first-order and pseudo-second-order models were applied in their linearized forms, as described in Eqs. 6 and 7, respectively.

6 $${q}_{t}= {q}_{e}- {q}_{e}{e}^{-{k}_{1}t}$$

7 $${q}_{t}=\frac{{k}_{2}{q}_{e}^{2} t}{{1+k}_{2}{q}_{e}t}$$

where *q*_*t*_ is the adsorption capacity at time *t* (mg g^−1^), *k*_1_ (min^−1^) and *k*_2_ (g mg^−1^ min^−1^) are the rate constants associated with pseudo-first-order and pseudo-second-order kinetics, respectively.

### Statistical analysis and model fitting

The adsorption isotherm and kinetic parameters were obtained by fitting the experimental data to the respective models using nonlinear regression analysis. The Langmuir and Freundlich isotherm models, as well as the pseudo-first-order (PFO) and pseudo-second-order (PSO) kinetic models, were applied to the experimental data by minimizing the residuals between calculated and experimental values. The goodness of fit of each model was evaluated using the coefficient of determination (*R*^2^). Higher *R*^2^ values were considered indicative of better agreement between the model predictions and experimental data.

All fittings and statistical analyses were performed using the software OriginPro, and the reported parameters correspond to the best-fit values obtained from the regression analysis. Each adsorption experiment was performed in triplicate, and the mean values were used for model fitting.

## Results and discussions

### Influence of pyrolysis temperature in dye removal

**Fig. 1:**
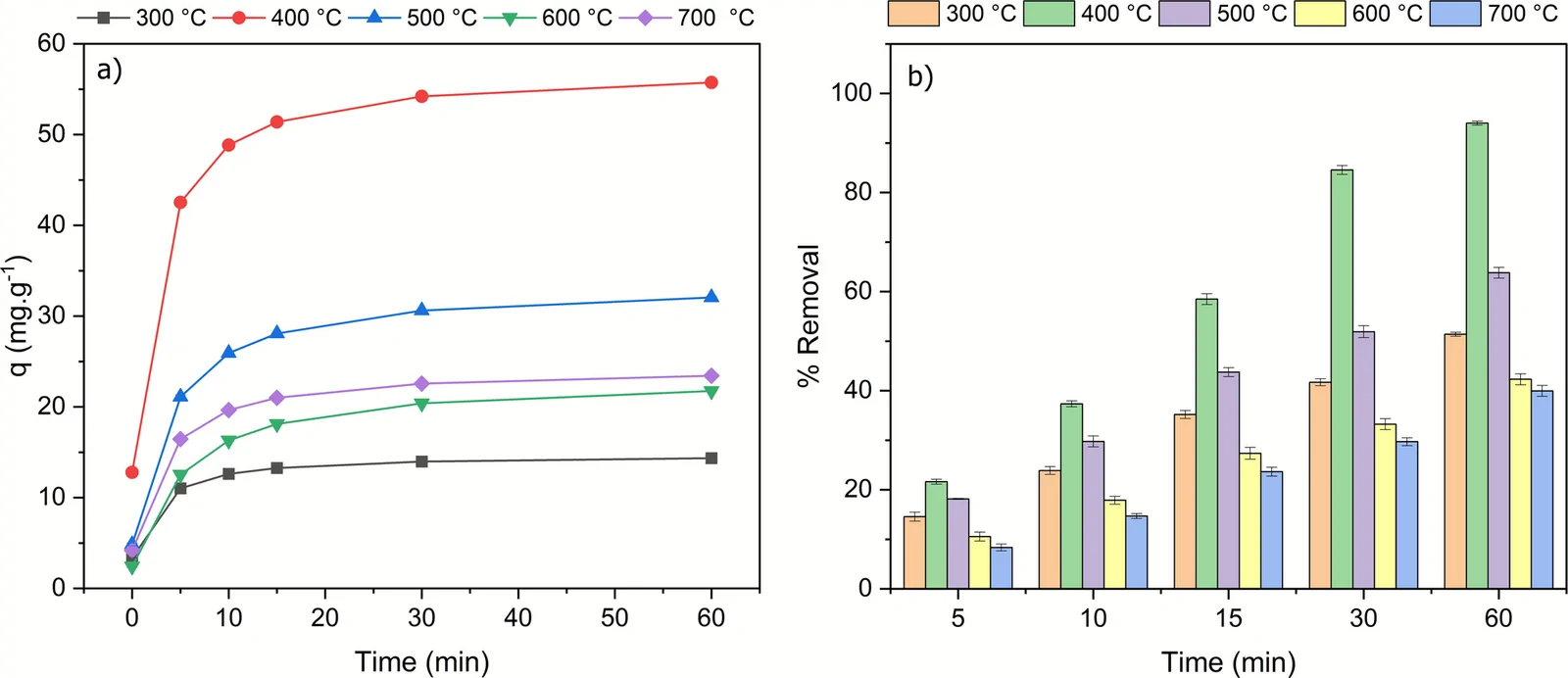
Adsorbed amounts (*q*) and the dye removal percentages (% removal) achieved by the tested biochars. In the *q* versus Time plot, the error bars are not visually distinguishable due to the very small experimental deviation (maximum standard deviation of 0.21 mg g^*−*1^)

Figure 1 presents the adsorbed amounts and the dye removal percentages achieved by the biochars produced at different pyrolysis temperatures, evaluated at an initial dye concentration of 100 mg L^−1^.

It can be observed that, for all biochars analyzed, most of the adsorption occurs within the first minutes, particularly during the initial 15 min. After 30 min, the adsorbed amounts vary only slightly, suggesting that equilibrium has been reached. This behavior is typical of systems in which a high availability of active sites is present at the beginning of the process, which is initially controlled by liquid-phase diffusion, followed by surface adsorption (Yamsomphong et al. 2025).

**Fig. 2:**
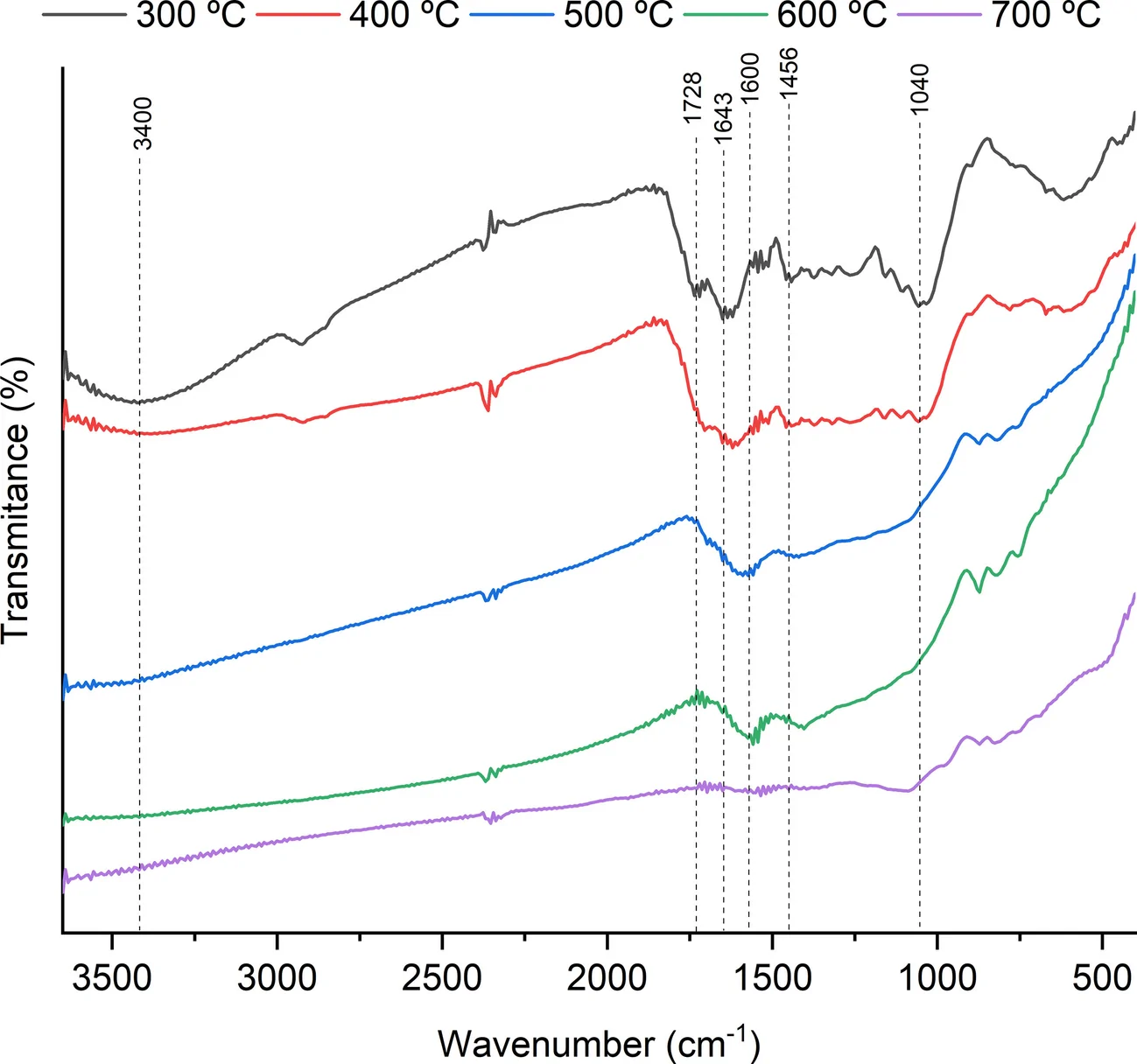
FTIR spectrum of biochar at different temperatures. Adapted from Silva et al. (2022)

After 60 min, the adsorbed amount of dye increases from 14.35 to 55.74 mg g^−1^ as the pyrolysis temperature rises from 300 to 400 °C, but then decreases to 32.05, 21.74, and 23.43 mg g^−1^ for the biochars produced at 500, 600, and 700 °C, respectively. A similar trend was observed for methylene blue removal, which increased from 51.4% (BC300) to 94% (BC400), followed by decreases to 63.8% (BC500), 42.3% (BC600), and 39.95% (BC700). This behavior can be explained by the materials’ characterization data. The FTIR spectrum (Fig. 2) provide insights into the surface chemistry, while the textural properties (surface area and pore volume) and elemental composition (carbon and oxygen content) are summarized in Table 1.

**Table 1:** Surface area, pore volume, carbon content, and oxygen content of the produced biochars (Silva et al. 2022)

Property	Pyrolysis temperature (°C)				
	300	400	500	600	700*
Surface area (m^2^ g^−1^)	9.81	60.51	112.8	272	349
Pore volume (cm^3^ g^−1^)	0.022	0.059	0.094	0.222	0.309
C	48.56	56.16	72.14	74.11	76.53
O	40.56	32.41	12.25	10.91	9.14
O/C	0.84	0.58	0.17	0.15	0.12

*Results obtained in the present study that have not been previously published

During the pyrolysis, the biochars surface area increases with temperature due to the formation of additional pores (Table 1). Simultaneously, the oxygen-rich functional groups, such as hydroxyls (3400 cm^−1^), carboxyls, and carbonyls (1728–1040 cm^−1^), which play a crucial role in dye adsorption due to their ability to form hydrogen bonds and electrostatic interactions (Sriram et al. 2025), are degraded at higher temperatures, as evidenced by the FTIR spectra and O/C ratio.

The evolution of the textural properties with increasing pyrolysis temperature provides a clear quantitative basis for the observed adsorption behavior. As shown in Table 1, the surface area increases sharply from 9.81 m^2^ g^−1^ for BC300 to 60.51 m^2^ g^−1^ for BC400, followed by a continuous rise to 349 m^2^ g^−1^ for BC700. A similar trend is observed for pore volume, which increases from 0.022 to 0.309 cm^3^ g^−1^ over the same temperature range. This progressive development of porosity can be attributed to the thermal decomposition of hemicellulose and cellulose at intermediate temperatures and the subsequent breakdown of lignin at higher temperatures, which promotes volatilization and pore formation (Silva et al. 2022). However, despite the substantial increase in surface area and pore volume at higher temperatures, the adsorption capacity does not follow the same monotonic trend, indicating that textural properties alone are insufficient to explain methylene blue uptake.

Elemental analysis further elucidates this discrepancy between textural development and adsorption performance. With increasing pyrolysis temperature, the carbon content rises from 48.56% (BC300) to 76.53% (BC700), while the oxygen content decreases markedly from 40.56 to 9.14%, resulting in a pronounced reduction in the O/C ratio from 0.84 to 0.12 (Table 1). This trend reflects the progressive elimination of oxygen-containing functional groups through dehydration, decarboxylation, and decarbonylation reactions during pyrolysis (Luo et al. 2015). The biochar produced at 400 °C exhibits an intermediate O/C ratio of 0.58, indicating the coexistence of developed porosity and a relatively high density of polar surface functionalities.

This finding highlights that adsorption performance is not determined exclusively by textural properties, but rather by a balance between porosity and surface chemistry. The oxygenated groups act as key active sites for dye binding and their thermal degradation leading to a decrease in adsorption efficiency even when more pores are available (Fan et al. 2018; Roy et al. 2023). In other words, a higher surface area without sufficient chemical functionality provides predominantly inert adsorption sites, which are less effective for specific interactions with cationic dyes such as methylene blue.

The evolution of these properties can be explained by the thermochemical decomposition of the lignocellulosic components of the acerola residue during pyrolysis. The biomass presents cellulose, hemicellulose, and lignin contents of 30.37 ± 0.11%, 19.95 ± 0.66%, and 18.10 ± 0.43%, respectively (Silva et al. 2022). Hemicellulose and cellulose decompose predominantly at lower temperatures (200–400 °C), releasing volatile compounds that promote initial pore formation, whereas lignin decomposes over a broader temperature range and contributes to the formation of more aromatic and thermally stable carbon structures (Hou et al. 2023). The relatively moderate lignin content of acerola residue favors pore development at intermediate temperatures while limiting excessive aromatization at higher temperatures, which helps explain the superior adsorption performance observed for the biochar produced at 400 °C.

A comparison with other biomass types reported in the literature further supports this interpretation. Herbaceous residues, such as rice straw, which typically contain higher hemicellulose contents, tend to undergo extensive devolatilization at lower pyrolysis temperatures, often producing biochars with moderate surface areas but limited structural stability (Bhardwaj et al. 2022). In contrast, woody biomasses, characterized by higher lignin contents (commonly above 25%), generally yield biochars with highly aromatic and graphitized structures when pyrolyzed at elevated temperatures, resulting in high surface areas but reduced concentrations of surface functional groups (Han et al. 2013). In this context, acerola residue exhibits an intermediate lignocellulosic profile, which leads to a trade-off between porosity development and functional group preservation that is strongly dependent on the pyrolysis temperature.

This compositional influence is consistent with the FTIR analysis (Fig. 2), which reveals a progressive reduction in bands associated with hydroxyl, carbonyl, and carboxyl groups as the pyrolysis temperature increases. These oxygen-containing functional groups are crucial for the adsorption of methylene blue, as they enable electrostatic interactions between negatively charged surface sites and the cationic dye molecules, as well as hydrogen bonding. At intermediate temperatures, such interactions are maximized due to the coexistence of accessible pores and abundant surface functionalities (Tan et al. 2015; Sriram et al. 2025).

The adsorption of methylene blue onto acerola residue-derived biochars is governed by a combination of mechanisms, including electrostatic attraction, hydrogen bonding, *π*–*π* interactions between the aromatic structures of the biochar and the dye molecules, and physical adsorption through pore filling (Liu et al. 2024). While higher pyrolysis temperatures may enhance *π*–*π* interactions due to increased aromaticity, the concurrent loss of polar functional groups diminishes electrostatic interactions, resulting in an overall decline in adsorption efficiency (Luo et al. 2015). This mechanistic balance explains why the highest adsorption capacity and removal efficiency were achieved at 400 °C, despite the continued increase in surface area at higher temperatures.

Thus, the results observed here demonstrate that the design of biochars for adsorption applications must consider not only structural parameters, such as porosity and surface area, but also strategies to preserve oxygenated surface sites. This underscores the importance of tailoring pyrolysis conditions to achieve an optimal balance between textural development and surface chemistry, particularly when targeting the removal of cationic organic dyes from aqueous systems.

### Adsorption equilibrium isotherms

Table 2 presents the parameters obtained from fitting the experimental data using adsorption isotherm models. The analyzed parameters show that the Langmuir model (0.91 < *R*^2^ < 0.98) provides a better description of the dye removal process than the Freundlich model (0.81 < *R*^2^ < 0.94). This suggests that methylene blue adsorption occurs predominantly through monolayer coverage on a finite number of energetically similar active sites. In this context, each site can adsorb only one dye molecule, and saturation is achieved when all available sites are occupied, resulting in a well-defined maximum adsorption capacity (Langmuir 1918).

**Table 2:** Biochar isothermal parameters adsorption

T (°C)	Langmuir				Freundlich		
	***q***_máx _mg g^−1^	***k***_L _L mg^−1^	***R***^2^	***R***_L_	***n***_F_	***k***_F _(mg g)^−1^ (L mg^−1^)^1/***n***^	***R***^2^
**300**	26.62	0.70	0.96	0.90	1.50	10.11	0.94
**400**	67.29	0.07	0.91	0.88	1.14	4.55	0.91
**500**	17.19	0.17	0.97	0.89	2.44	4.01	0.89
**600**	11.12	0.15	0.94	0.91	3.17	3.12	0.89
**700**	17.12	0.07	0.98	0.84	1.99	2.21	0.81

Although biochars are intrinsically heterogeneous materials, the superior performance of the Langmuir model indicates that methylene blue adsorption is mainly governed by specific surface sites with comparable adsorption energies (Langmuir 1918). These dominant sites are largely associated with oxygen-containing functional groups identified by FTIR analysis in the previous section, which act as preferential binding centers for cationic dye molecules (Bhardwaj et al. 2022). Once these chemically active sites are saturated, further adsorption becomes limited, leading to the observed monolayer behavior despite the inherent heterogeneity of the biochar surface.

The analysis of the parameters presented in Table 2 reveals a complex and non-linear relationship between pyrolysis temperature and adsorption capacity, reflecting the competing influence of textural properties and surface chemistry. The maximum adsorption capacity (*q*_max_) increases from 26.62 to 67.29 mg g^−1^ as the pyrolysis temperature rises from 300 to 400 °C. However, with further temperature increases, *q*_max_ decreases significantly, reaching 17.12 mg g^−1^ for the biochar produced at 700 °C. This trend is consistent with the results discussed previously, where an optimal balance between pore development and preservation of oxygenated functional groups was observed at intermediate pyrolysis temperatures.

Similar behavior has been reported in the literature. Güleç et al. (2022) observed superior methylene blue adsorption efficiencies for biochars derived from rapeseed and whitewood produced at relatively low thermal conversion temperatures (250–300 °C). In their study, the Langmuir model also provided the best fit to the experimental data, with *q*_max_ values of approximately 60 mg g^−1^ and 30 mg g^−1^ for rapeseed and whitewood biochars, respectively. The authors attributed this performance to the higher abundance of surface functional groups at lower temperatures, noting that insufficient thermal severity prevents extensive cracking of lignocellulosic bonds and thus preserves adsorption-active functionalities, even in materials with comparatively lower surface areas.

In addition to *q*_max_, the Langmuir constant (*k*_L_), which is related to the affinity between the adsorbent and the adsorbate, provides further insight into the adsorption mechanism (Langmuir 1918). The lower *k*_L_ values observed for biochars produced at higher pyrolysis temperatures indicate weaker interactions between methylene blue molecules and the biochar surface (Liu 2009). This behavior can be attributed to the progressive loss of oxygenated functional groups and the concomitant reduction in electrostatic attraction, reinforcing the conclusion that surface chemistry plays a more decisive role than surface area in controlling adsorption performance.

The separation factor (*R*_L_) values obtained for all biochars fall within the range 0 < *R*_L_ < 1, indicating that MB adsorption is favorable across the entire range of pyrolysis temperatures investigated. However, variations in *R*_L_ values reflect differences in interaction strength, with more favorable adsorption behavior associated with biochars produced at lower and intermediate temperatures, in agreement with the trends observed for *q*_max_ and *k*_L_.

Although the Freundlich model yielded slightly lower correlation coefficients, it still provided a satisfactory fit to the experimental data, confirming the heterogeneous nature of the biochar surfaces. The Freundlich intensity parameter (*n*_F_) was greater than unity for all samples, indicating favorable adsorption (Foo and Hameed 2010) . Notably, the biochar produced at 400 °C, which exhibited the highest adsorption capacity, showed the lowest *n*_F_ value (1.14), suggesting a highly heterogeneous surface energy distribution that favors the coexistence of diverse active sites capable of interacting with methylene blue molecules (Gao et al. 2025).

In contrast, the significantly higher *n*_F_ values observed for the 500 °C and 600 °C biochars (2.44 and 3.17, respectively) indicate a transition toward a more energetically uniform surface, associated with increased carbonization and structural rearrangement at elevated temperatures. This shift toward homogeneity occurs alongside a marked decrease in overall adsorption capacity, as reflected in the lower *k*_F_ and *q*_max_ values, highlighting that surface heterogeneity combined with abundant functional groups is a key driver for efficient methylene blue removal in this system (Tan et al. 2015).

Overall, the isotherm analysis corroborates the mechanistic interpretation presented in the previous section, demonstrating that the adsorption behavior of acerola residue-derived biochars is governed primarily by the availability, distribution, and chemical nature of surface functional groups rather than by surface area alone. The strong dependence of isotherm parameters on pyrolysis temperature further emphasizes the importance of tailoring thermal conversion conditions to achieve an optimal balance between structural development and surface chemistry for dye adsorption applications.

### Adsorption kinetics

Table 3 summarizes the kinetic parameters obtained for methylene blue adsorption onto the produced biochars at different initial dye concentrations.

**Table 3:** Biochar kinetic model parameters

300 °C MB mg L^−1^	Pseudo-first-order			Pseudo-second-order		
	***k***_1 _min^−1^	***q***_***e ***_mg g^−1^	***R***^2^	***k***_2 _g mg^−1^ min^−1^	***q***_***e ***_mg g^−1^	***R***^2^
10	0.39	1.80	0.94	0.27	1.97	0.87
30	0.45	0.93	0.92	0.60	1.01	0.83
50	0.37	4.69	0.95	0.10	5,17	0.90
100	0.39	13.46	0.95	0.04	14.76	0.88
150	0.33	7.77	0.96	0.05	8.57	0.92
**400 °C** **MB mg L**^**−1**^	**Pseudo-first-order**			**Pseudo-second-order**		
	***k***_**1**_ **min**^**−1**^	***q***_***e***_ **mg g**^**−1**^	***R***^**2**^	***k***_**2**_ **g mg**^**−1**^** min**^**−1**^	***q***_***e***_ **mg g**^**−1**^	***R***^**2**^
10	0.13	2.25	0,90	0.05	2.66	0.87
30	0.39	8.21	0.94	0.05	9.06	0.89
50	0.35	14.17	0.96	0.03	15.64	0.91
100	0.21	51.23	0.98	0.01	57.36	0.96
150	0.10	92.20	0.99	0.00	109.8	0.98
**500 °C** **MB mg L**^**−1**^	**Pseudo-first-order**			**Pseudo-second-order**		
	***k***_**1**_ **min**^**−1**^	***q***_***e***_ **mg g**^**−1**^	***R***^**2**^	***k***_**2**_ **g mg**^**−1**^** min**^**−1**^	***q***_***e***_ **mg g**^**−1**^	***R***^**2**^
10	-	-	-	-	-	-
30	0.42	3.17	0.93	0.16	3.48	0.86
50	0.35	6.20	0.96	0.07	6.84	0.91
100	0.35	30.19	0.95	0.01	33.64	0.91
150	0.16	44.52	0.96	0.004	51,23	0.96
**600 °C** **MB mg L**^**−1**^	**Pseudo-first-order**			**Pseudo-second-order**		
	***k***_**1**_ **min**^**−1**^	***q***_***e***_ **mg g**^**−1**^	***R***^**2**^	***k***_**2**_ **g mg**^**−1**^** min**^**−1**^	**q**_**e**_ **mg g**^**−1**^	***R***^**2**^
10	-	-	-	-	-	-
30	0.14	1.06	0.99	0.14	1.22	0.97
50	0.33	9.88	0.96	0.04	10.97	0.92
100	0.20	20.53	0.96	0.01	23.30	0.96
150	0.07	30.93	0,95	0.002	38.30	0.93
**700 °C** **MB mg L**^**−1**^	**Pseudo-first-order**			**Pseudo-second-order**		
	***k***_**1**_ **min**^**−1**^	***q***_***e***_ **mg g**^**−1**^	***R***^**2**^	***k***_**2**_ **g mg**^**−1**^** min**^**−1**^	***q***_***e***_ **mg g**^**−1**^	***R***^**2**^
10	-	-	-	-	-	-
30	0.06	0.38	0.90	0.11	0.50	0.91
50	0.04	4.54	0.95	0.004	7,24	0.95
100	0.30	22.10	0.97	0.017	24.38	0.92
150	0.14	35.49	0.95	0.005	40.03	0.91

Regarding adsorption kinetics (Table 3), it was observed that equilibrium was reached rapidly for all produced biochars, regardless of pyrolysis temperature. In all experiments, a substantial fraction of methylene blue was adsorbed within the first 30 s, evidencing the fast nature of the process. Additionally, the equilibrium adsorption capacity (*q*_*e*_) increased systematically with increasing initial MB concentration, indicating that higher concentration gradients enhance mass transfer from the solution to the biochar surface (Deng et al. 2011).

The rapid initial uptake can be attributed to the high availability of active adsorption sites at the beginning of the process, combined with strong electrostatic interactions between the cationic dye molecules and oxygen-containing functional groups on the biochar surface. Furthermore, physical adhesion within the pore network contributes to the fast adsorption rate, as previously reported for similar biochar-based systems (Deng et al. 2011; Bhagavathi et al. 2022). As adsorption progresses, the gradual occupation of active sites leads to a decrease in the adsorption rate until equilibrium is reached.

Both the pseudo-first-order (PFO) and pseudo-second-order (PSO) kinetic models provided satisfactory fits to the experimental data, as indicated by the relatively high determination coefficients (*R*^2^). However, the PFO model consistently yielded slightly higher *R*^2^ values across most conditions, suggesting a better overall representation of the adsorption process. This indicates that MB adsorption onto acerola residue-derived biochars is predominantly controlled by diffusion-driven mass transfer toward available surface sites rather than by surface reaction kinetics alone (Bhagavathi et al. 2022).

According to the PFO model, the adsorption rate is proportional to the number of unoccupied active sites, which explains the rapid adsorption observed at the early stages of the batch experiments. As these sites become progressively filled, the adsorption rate decreases, leading to equilibrium. This behavior is characteristic of systems where physisorption and external or intraparticle diffusion play a significant role in governing the overall adsorption process (Al-Asadi et al. 2025).

A critical trend observed in Table 3 is the systematic increase in experimentally determined *q*_*e*_ values with increasing initial MB concentration, a behavior consistently predicted by both kinetic models. This effect is driven by the increased driving force for mass transfer: higher initial dye concentrations generate steeper concentration gradients between the bulk solution and the biochar surface, facilitating faster diffusion and allowing a greater number of dye molecules to occupy available adsorption sites (Ge et al. 2023).

Among all samples, the biochar produced at 400 °C exhibited a remarkably superior adsorption performance. At an initial MB concentration of 150 mg L^−1^, this material achieved an equilibrium adsorption capacity of 92.20 mg g^−1^, significantly outperforming biochars produced at other temperatures. This result is fully consistent with the isotherm analysis and reinforces the conclusion that pyrolysis at 400 °C optimally tailors the biochar surface, preserving a high density of oxygenated functional groups while maintaining sufficient pore accessibility. These surface characteristics favor strong interactions with MB molecules, likely involving a combination of electrostatic attraction and surface complexation (Li et al. 2016; Fan et al. 2018).

In contrast, biochars produced at higher pyrolysis temperatures (500–700 °C) exhibited markedly lower adsorption capacities, despite sometimes showing good kinetic model fits. This behavior supports the hypothesis established in the isotherm discussion: increasing pyrolysis temperature promotes carbonization and structural homogenization, while simultaneously degrading oxygen-containing functional groups that are essential for effective MB adsorption. As a result, adsorption in these high-temperature biochars becomes increasingly dominated by weaker physical interactions, as reflected by the continued suitability of the PFO model and the reduced *q*_*e*_ values (Ambaye et al. 2021).

Overall, the kinetic study confirms that methylene blue adsorption onto acerola residue-derived biochars is a fast and diffusion-controlled process, strongly influenced by the initial dye concentration and the surface chemistry induced by pyrolysis temperature. The outstanding performance of the 400 °C biochar highlights the importance of achieving an optimal balance between porous structure and chemically active surface sites, a balance that is progressively lost at higher thermal conversion temperatures.

## Conclusions

Biochars produced from the pyrolysis of acerola industrial residue at various temperatures were evaluated as adsorbents for methylene blue dye. The Langmuir model best fit the data, indicating monolayer adsorption on a uniform surface. The maximum adsorption capacity increased significantly from 300 to 400 °C, reaching 67.29 mg g^−1^, but decreased at higher temperatures due to the degradation of oxygenated functional groups. The biochar produced at 400 °C demonstrated the best balance between surface area and functional group preservation. These findings underscore the potential of this biochar as an effective and sustainable material for effluent treatment.

## Data Availability

The datasets generated and analyzed during the current study are available from the corresponding author upon reasonable request. In addition, some data related to the characterization of the biochars were obtained from a previously published article, which has been properly cited in the manuscript: Silva JDO, Wisniewski JA, Carregosa ISC, Silva WR, Abud AKS, Oliveira Júnior AM (2022) Thermovalorization of acerola industrial waste by pyrolysis in a continuous rotary kiln reactor. J Anal Appl Pyrol 161: 105,373. https://doi.org/10.1016/j.jaap.2021.105373.
